# Prevalence and implications of bilateral and solely contralateral lymph node metastases in oral squamous cell carcinoma

**DOI:** 10.1007/s00784-024-05650-1

**Published:** 2024-04-23

**Authors:** Ann-Kristin Struckmeier, Mayte Buchbender, Abbas Agaimy, Marco Kesting

**Affiliations:** 1 Department of Oral and Cranio-Maxillofacial Surgery, Friedrich-Alexander-Universität Erlangen- Nürnberg (FAU), Glückstraße 11, 91054 Erlangen, Germany; 2 Comprehensive Cancer Center Erlangen-European Metropolitan Area of Nuremberg (CCC ER- EMN), Erlangen, Germany; 3 Institute of Pathology, Friedrich-Alexander-Universität Erlangen-Nürnberg (FAU), Erlangen, Germany

**Keywords:** Lymph node metastasis, Neck dissection, Bilateral metastases, Contralateral metastases, Oral squamous cell carcinoma

## Abstract

**Objectives:**

Effective management of neck in oral squamous cell carcinoma (OSCC) is pivotal for oncological outcomes. Although consensus exists for ipsilateral neck dissection (ND), the necessity for contralateral ND remains controversial. This study aimed to assess the prevalence and implications of bilateral/solely contralateral (B/SC) lymph node metastases (LNMs) to determine the need for contralateral elective ND. Additionally, it examined the prevalence and implications of occult B/SC metastases.

**Materials and methods:**

In a retrospective cohort study, 420 OSCC patients underwent primary surgical treatment following German guidelines at a tertiary center. Preoperative contrast-enhanced computed tomography was conducted, and ND adhered to a standardized approach.

**Results:**

Solely contralateral metastases occurred in 0.95% of patients, with bilateral metastases observed in 7.13%. Occult B/SC metastases occurred in 3.81% of the cases. Correlation analysis revealed a statistically significant association between B/SC metastases and higher tumor stages, tumor localization at the upper jaw or floor of the mouth, proximity to the midline, ipsilateral LNMs, and lymphatic invasion (all *p* < 0.05). Patients with B/SC metastases showed poorer disease-free survival, with statistical significance reached in the bilateral LNMs group (*p* = 0.010). Similarly, a significant difference was noted in overall survival between patients with bilateral and solely ipsilateral metastases (*p* = 0.044).

**Conclusions:**

B/SC LNMs are rare in patients with OSCC, especially in those who present with clinico-radiologically negative ipsilateral necks. Higher rates of B/SC metastases occur in case of advanced tumors and those localized at the upper jaw or floor of the mouth. Ipsilateral LNMs significantly elevate the risk of contralateral LNMs, tripling the associated risk.

**Clinical relevance:**

These findings provide valuable insights for surgeons considering contralateral ND or extended adjuvant treatment for OSCC patients. However, the absence of high-level evidence from randomized controlled trials impedes the establishment of a definitive standard of care.

**Supplementary Information:**

The online version contains supplementary material available at 10.1007/s00784-024-05650-1.

## Introduction

Oral squamous cell carcinoma (OSCC) exhibits a substantial risk for cervical lymph node metastases (LNMs), impacting around 42.6% of patients [1]. Given that nodal dissemination holds pivotal prognostic significance in OSCC patients [2], effective neck management is imperative for oncologic control and survival [3]. While established consensus and guidelines exist for ipsilateral neck dissection (ND) in OSCC [4], the approach to contralateral neck management remains contentious.

Elective treatment of the contralateral neck is generally accepted for midline-invading OSCC but is not routine in lateralized cases. The incidence of B/SC metastases varies widely, ranging from 0.9 to 36% in previous studies [5].

B/SC metastases occurs through various mechanisms, including crossing afferent lymphatic vessels, tumor spread along the midline, particularly in cases with extensively involved ipsilateral lymph nodes, and in anatomical areas lacking a distinct midline barrier [6]. Especially, the floor of the mouth is more frequently associated with contralateral metastases compared to other sites, such as the anterior part of the tongue and the retromolar region [6] due to the extensive submucosal lymphatic plexus, freely communicating and crossing the midline [7].

Rates of contralateral neck failure have been reported at 3–17% in multiple case series [8–10]. Hence, conducting a thorough preoperative assessment to predict the likelihood of LNMs is imperative for precise planning of ND. This is crucial because the development of clinically detectable LNMs is linked with diminished oncological outcomes [11]. On the contrary, extending ND to the contralateral side without clinical evidence of LNMs may lead to additional morbidity, including shoulder pain and dysfunction resulting from accessory nerve paralysis [12].

The aim of this study was to ascertain the prevalence and risk factors associated with B/SC metastases to evaluate the necessity for contralateral elective ND (END), and to explore whether their presence influences disease-free survival (DFS) and overall survival (OS). Additionally, we investigated the percentage and implications of occult B/SC metastases.

## Methods

### Study design and participants

The study cohort encompassed patients with primary OSCC, who received treatment including radical tumor resection and ND. The treatment protocol followed the established German guidelines and patients were treated at a high-volume tertiary medical center from January 1, 2013, to May 31, 2023. All therapeutic interventions were in accordance with the recommendations made during oncology board meetings.

At our tertiary medical center, the primary surgical approach for managing OSCC entails radical tumor resection, often supplemented with reconstruction using a free flap when deemed necessary, along with ND. ND is systematically carried out in every patient, following a well-established protocol. For those without LNMs, we perform an ipsilateral selective ND (SND) covering levels I to III, commonly known as supraomohyoid ND. In cases where tumors are located at or approaching the midline, a bilateral SND is undertaken.

When there are preoperative, intraoperative (utilizing the frozen section technique), or postoperative proofs of ipsilateral LNMs, we conduct a modified radical ND (MRND) on the ipsilateral side, accompanied by a contralateral SND. If bilateral LNMs are present, a bilateral MRND is performed. Our consistent practice involves split up ND, where lymph node specimens are dissected into packages, enabling the categorization of LNMs into cervical levels following histopathological analysis.

The decision to administer adjuvant radiotherapy or radiochemotherapy was made by assessing the individual risk factors of each patient, aligning with the guidelines outlined in the German protocol.

The exclusion criteria included recurrent OSCC and squamous cell carcinoma of the lip. Patients who declined ND or underwent a limited ND due to severe comorbidities were also excluded. To mitigate potential bias arising from surgery-related short-term mortality, individuals who passed away within 30 days following surgery (perioperative death) were excluded from survival analyses. Additionally, patients with a follow-up period of less than 30 days were not included in the study.

The design and methodologies of the study obtained approval from the Ethics Committee of Friedrich-Alexander-University Erlangen-Nuremberg (Ethic votes: 23-185-Br, 23-186-Br). In adherence to national and institutional regulations, written informed consent was deemed unnecessary.

The manuscript was drafted in accordance with the STROBE (Strengthening the Reporting of Observational Studies in Epidemiology) statement.

### Follow-up schedule

The follow-up schedule was structured as follows: clinical examinations were carried out every 6 weeks in the first year, shifted to every 3 months in the second and third years, and continued with 6-month intervals during the fourth and fifth years. CT scans were additionally performed every 6 months during the initial two years and transitioned to a 12-month frequency in the following three years.

### Contrast-enhanced computed tomography

Before undergoing surgery, all participants in this study underwent preoperative staging through thin-section axial multidetector CT scans. These scans utilized intravenous iodine-based contrast agents to enhance the differentiation of soft tissues. The evaluation of imaging data was performed by a minimum of two independent physicians from the Department of Radiology. At least one consultant assessed the local extent of the tumor and bone invasion.

### Clinicopathological characteristics

Clinicopathological characteristics were extracted from the medical records. The following parameters were methodically documented and analyzed: age, gender, tumor localization, tumor, node, metastasis (TNM) classification, localization of LNMs, depth of invasion (DOI), histological grading, resection margins, presence of perineural, vascular, and lymphatic invasion. Additionally, the time point of surgery, the last follow-up, and the time point of death were recorded.

The TNM classification underwent revision throughout the study period. For result consistency [13], patients initially categorized under the 7th TNM classification were restaged. Consequently, all patients were reclassified in accordance with the 8th TNM classification.

### Statistical analysis

Statistical analysis was conducted using the Statistical Package for the Social Sciences 28.0 (SPSS, Chicago, IL, USA).

Descriptive statistics were represented through frequency tables, cross tables, and bar charts. Categorical variables were expressed as absolute and relative frequencies. Relationships between different characteristics were determined using cross tables, with the probabilities of correlations checked through the chi-square test. Patient survival was analyzed and depicted using Kaplan–Meyer curves. We employed the log-rank test for comparing survival between groups.

A p value < 0.05 was considered statistically significant.

Figures were generated using SPSS.

## Results

### Patient cohort

The majority of patients in the cohort were male, constituting 258 individuals (61.87%). The mean age of the patient cohort was 64.75 (standard deviation: 12.03). Predominant tumor localizations included the floor of the mouth (147 patients, 35.25%) and the tongue (105 patients, 25.18%).

Pathological tumor stages were distributed as follows: T1 152 patients (36.45%), T2 106 patients (25.42%), T3 51 patients (12.23%), and T4a 108 patients (25.90%). Histopathological examination indicated the absence of LNMs in 275 patients (65.95%), while 44.05% presented with metastatic disease.

Approximately half of the patients had moderately differentiated carcinomas (51.32%, 214 patients), while 37.41% exhibited poorly differentiated carcinomas (156 patients), and only 9.52% displayed well-differentiated carcinomas (40 patients). Additionally, the analysis revealed lymphatic invasion in 8.15% (34 patients), vascular invasion in 2.40% (10 patients), and perineural invasion in 19.66% of the tumors (82 patients). Microscopically positive margins were observed in 1.92% of cases (8 patients).

Among these patients, 255 out of 420 (60.71%) received adjuvant treatment, encompassing brachytherapy, radiation, or radiochemotherapy. 29 patients (6.90%) either chose to forgo adjuvant therapy or did not complete it, despite its recommendation.

### Occurrence of bilateral and solely contralateral metastases

The investigation into the occurrence of B/SC metastases yielded the following results: Within our study group, the majority of metastases of the contralateral neck were associated with concurrent ipsilateral metastases, accounting for 7.13% (30 out of 420 cases). The likelihood of developing contralateral metastases without ipsilateral involvement was determined to be 0.95% (4 patients out of 420). Results are displayed in Table 1.

**Table 1:** Localization of lymph node metastases according to histopathological assessment

Localization of LNMs	Number (%)	Occult percentage (%)
Ipsilateral	110 (26.13)	
Bilateral	30 (7.13)	14 (3.33)
Solely contralateral	4 (0.95)	2 (0.48)

### Occult bilateral and solely contralateral metastases

Following that, we delved into the occurrence of occult metastases in the staging process, where either no LNMs were initially identified, or LNMs initially detected ipsilaterally were later revealed to be contralateral or bilateral.

In the preoperative assessment, CT unveiled bilateral metastases in 35 patients (8.31%) and contralateral metastases in 2 patients (0.48%). A certain number of patients (19 patients) were erroneously diagnosed with bilateral metastases, while another group of patients (16 out of 420 patients) who later exhibited bilateral metastases upon histological assessment were not initially identified with bilateral LNMs in CT. Data regarding the occurrence of occult LNMs are depicted in Tables 1 and 2.

**Table 2:** Localization of lymph node metastases according to computed tomography

Localization of LNMs	Number (%)
Ipsilateral	86 (20.43)
Bilateral	35 (8.31)
Solely contralateral	2 (0.48)

### Correlation between clinicopathological characteristics and bilateral/contralateral metastases

**Fig. 1:**
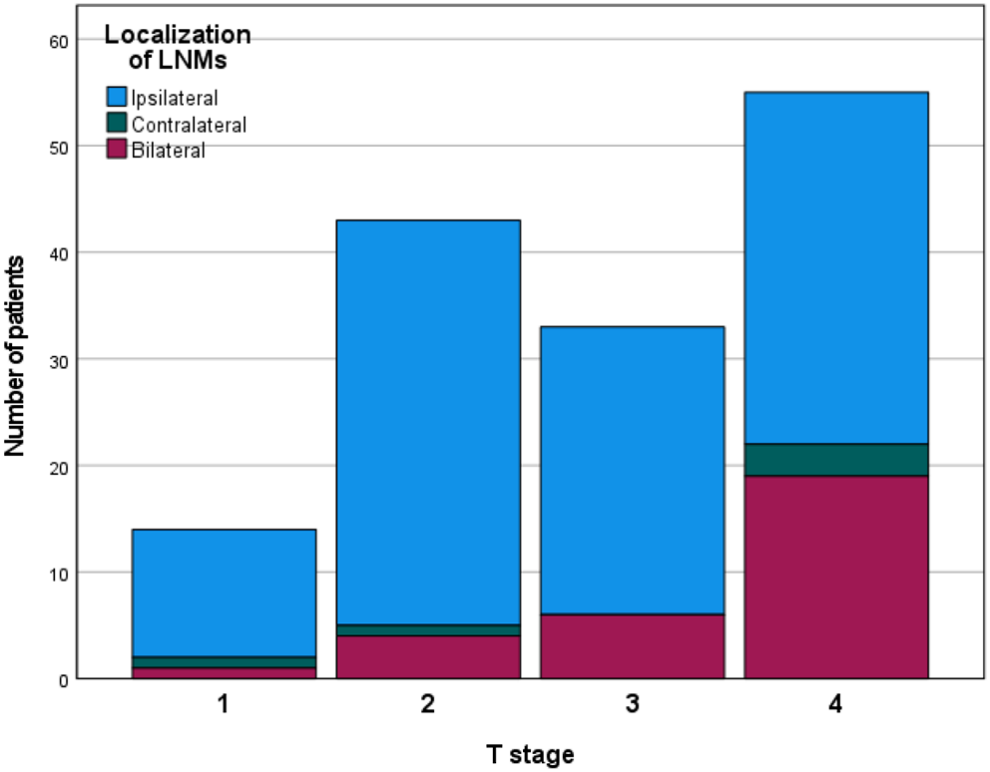
Localization of lymph node metastases depending on tumor stage

**Fig. 2:**
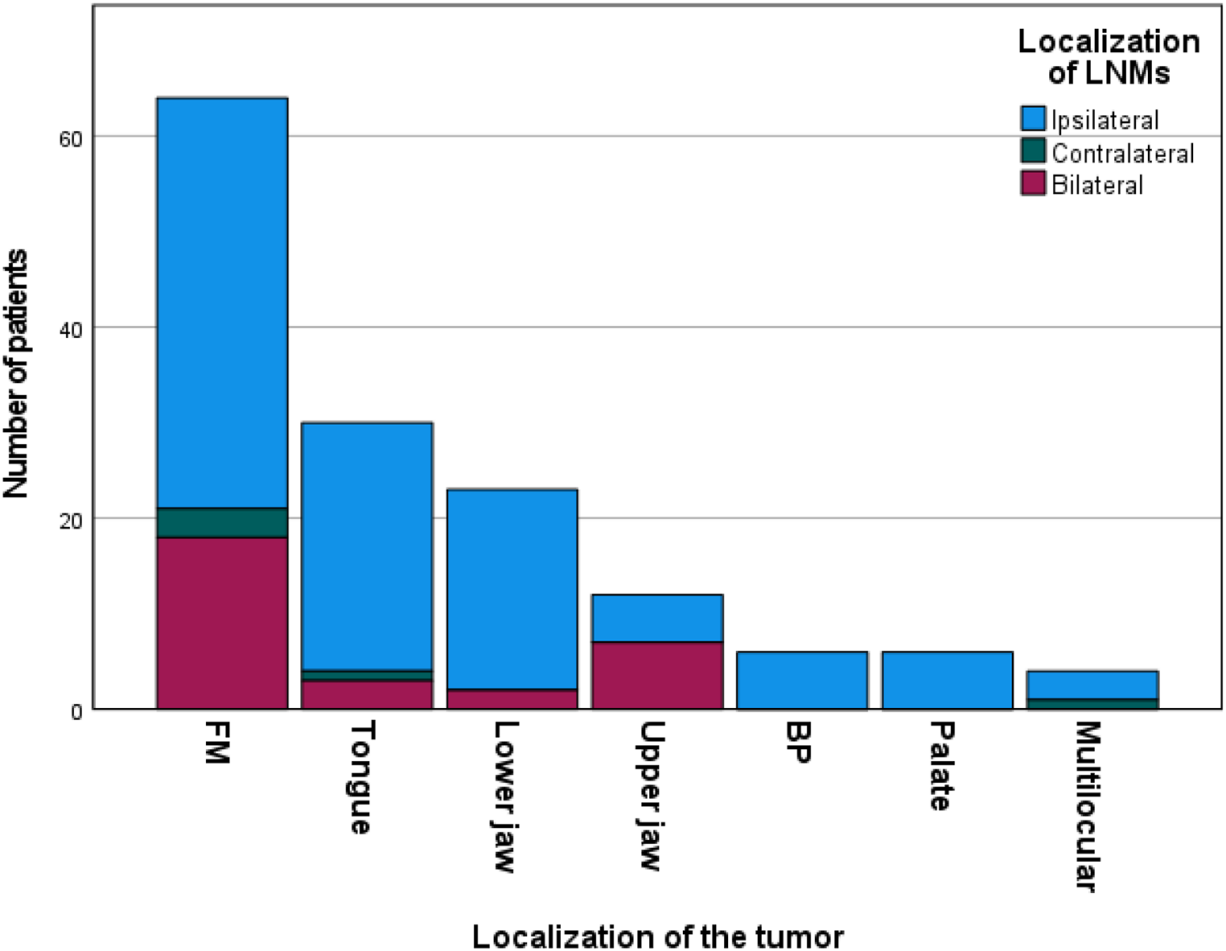
Localization of lymph node metastases depending on the localization of the tumor

Correlation analysis unveiled a statistically significant association between the occurrence of B/SC metastases and several factors: higher tumor stages (*p* = 0.009), tumor localization at the upper jaw or floor of the mouth (*p* = 0.005), lymphatic invasion (*p* = 0.016), the presence of ipsilateral LNMs (*p* = 0.005), and proximity to the midline (*p* = 0.011). As a result, the risk of B/SC metastases was notably elevated in T4a tumors (38.89% compared to < 20% in T1-T3). Additionally, patients with tumors demonstrating lymphatic invasion exhibited a heightened propensity for B/SC metastases (19.09% vs. 39.39%). Moreover, individuals with tumors located at the floor of the mouth (31.75%) or the upper jaw (58.33%) had a substantially higher likelihood of presenting with B/SC metastases. Unsurprisingly, tumors situated in the median or bilateral positions were more predisposed to exhibit B/SC metastases (22.73% vs. 60.71%). The results of the correlation analysis are displayed in Table 3. Furthermore, the results are graphically displayed in Figs. 1 and 2.

**Table 3:** Percentages of ipsilateral and contralateral/bilateral metastases depending on clinicopathological characteristics

Clinicopathological characteristics		Ipsilateral metastases (%)	Contralateral/ bilateral metastases (%)	Correlation (Chi-square)
Tumor stage	T1	12 (85.71)	2 (14.29)	0.009*
	T2	38 (88.37)	5 (11.63)	
	T3	27 (81.82)	6 (18.18)	
	T4a	33 (61.11)	21 (38.89)	
Sex	Male	69 (75.00)	23 (25.00)	0.602
	Female	41 (78.85)	11 (21.15)	
Age	< 65 years	35 (85.37)	6 (14.63)	0.325
	≥ 65 years	26 (76.47)	8 (23.53)	
Lymphatic invasion	L0	89 (80.91)	21 (19.09)	0.016*
	L1	20 (60.61)	13 (39.39)	
Vascular invasion	V0	104 (77.61)	30 (22.39)	0.132
	V1	5 (55.56)	4 (44.44)	
Perineural invasion	Pn0	74 (80.43)	18 (19.57)	0.112
	Pn1	35 (68.63)	16 (31.37)	
Grading	G1	1 (100.00)	0 (0.00)	0.673
	G2	41 (73.21)	15 (26.79)	
	G3	65 (78.31)	18 (21.69)	
Depth of invasion	≤ 5 mm	20 (76.92)	6 (23.08)	0.147
	6–10 mm	43 (87.76)	6 (12.24)	
	≥ 11 mm	42 (72.41)	16 (27.59)	
Localization	Floor of the mouth	43 (68.25)	20 (31.75)	0.005*
	Tongue	26 (86.67)	4 (13.33)	
	Lower jaw	21 (91.30)	2 (8.70)	
	Upper jaw	5 (41.67)	7 (58.33)	
	Buccal plane	6 (100.00)	0 (0.00)	
	Palate	6 (100.00)	0 (0.00)	
	Multilocular	3 (75.00)	1 (25.00)	
Proximity to the midline	Unilateral	68 (77.27)	20 (22.73)	0.011*
	Median/ bilateral	22 (39.29)	34 (60.71)	

### Survival analysis for patients with bilateral/solely contralateral metastases

We performed a survival analysis to compare outcomes among patients with ipsilateral metastases, solely contralateral metastases, and bilateral metastases. Survival outcomes were notably inferior in the latter two groups. However, regarding DFS, statistical significance was only observed in the bilateral metastases group (log-rank, *p* = 0.010) in comparison to ipsilateral metastases. Similarly, a significant difference was noted between bilateral metastases and ipsilateral metastases regarding OS (log-rank, *p* = 0.044). However, the disparity between contralateral metastases and ipsilateral metastases was nearly significant (log-rank, *p* = 0.086).

**Fig. 3:**
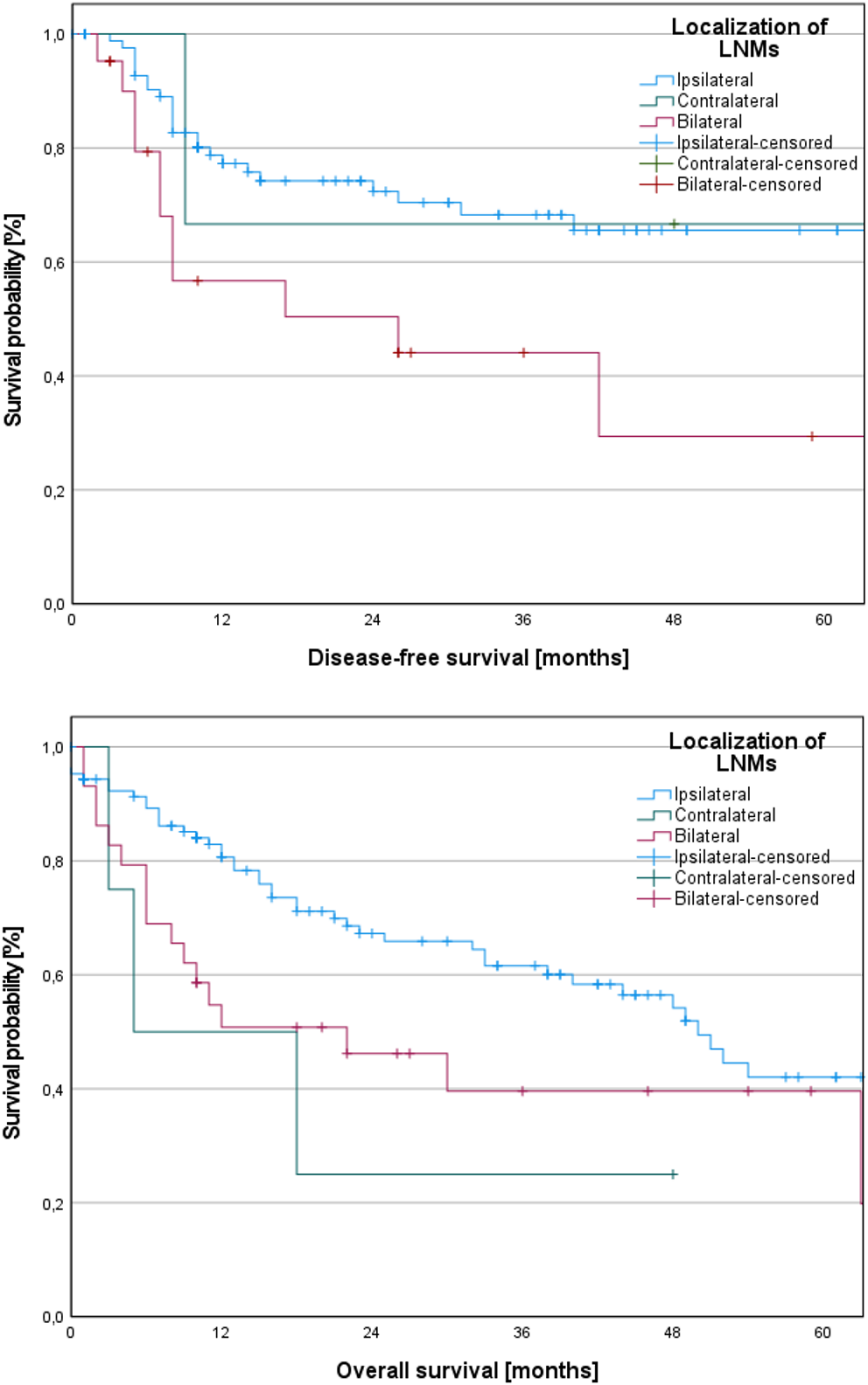
Kaplan-Meier curves of disease-free survival (DFS) and overall survival (OS) depending on the localization of lymph node metastases (LNMs). Patients with bilateral/solely contralateral metastases showed inferior survival outcomes. However, statistical significance was only reached in the bilateral metastases group regarding DFS (log-rank, *p* = 0.010). Similarly, a significant difference was noted between bilateral metastases and ipsilateral metastases in OS (log-rank, *p* = 0.044). Additionally, the disparity between contralateral metastases and ipsilateral metastases was nearly significant (log-rank, *p* = 0.086)

The corresponding Kaplan-Meier curves are depicted in Fig. 3.

### Survival analysis for patients with occult bilateral/solely contralateral metastases

Patients with occult B/SC metastases demonstrated notably poorer DFS in contrast to those without occult B/SC metastases (log-rank test, *p* = 0.040). Although there was an observable trend toward worse OS among patients with occult B/SC metastases, the disparity in OS between both groups did not reach statistical significance (log-rank, *p* = 0.099).

**Fig. 4:**
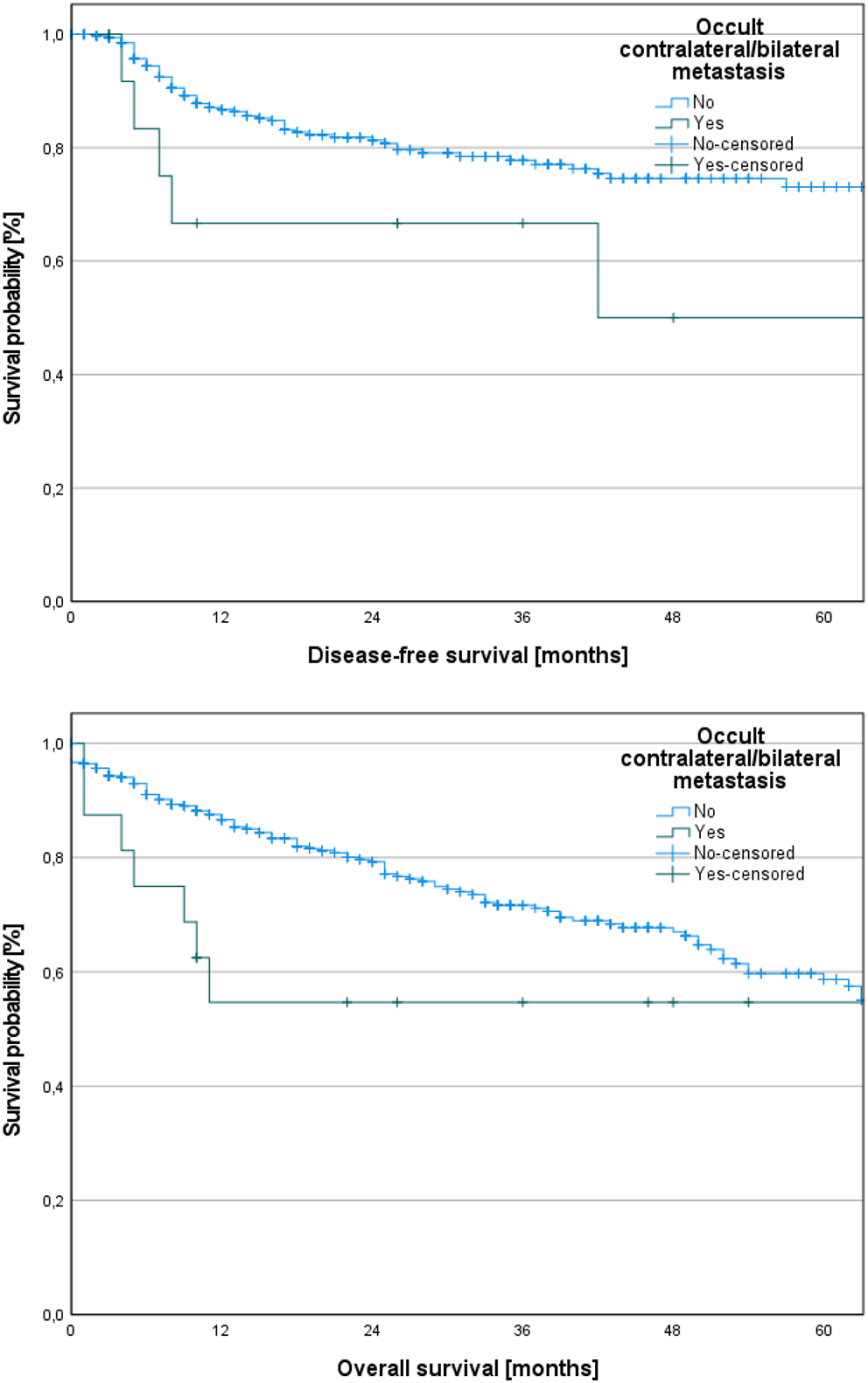
Kaplan-Meier curves of disease-free survival (DFS) and overall survival (OS) depending on the occurrence of occult bilateral/solely contralateral (B/SC) metastases. Patients with occult B/SC demonstrated notably poorer DFS in contrast to those without occult B/SC metastases (log-rank, *p* = 0.040). Although there was an observable trend toward worse OS among patients with occult B/SC metastases, the disparity in OS between both groups did not reach statistical significance (log-rank, *p* = 0.099)

The corresponding Kaplan-Meier curves of DFS and OS depending on the occurrence of B/SC metastases are displayed in Fig. 4.

## Discussion

OSCC patients exhibit a high propensity for cervical LNMs, typically appearing ipsilaterally but occasionally manifesting bilaterally or solely contralaterally. However, the existing literature on the rates of B/SC metastases and their associated risk factors are scarce. This study aimed to achieve two primary objectives: first, to identify the prevalence and risk factors associated with B/SC metastases, evaluating the necessity for contralateral END; and second, to explore whether their presence influences survival. Furthermore, we investigated the prevalence and implications of occult B/SC metastases.

The literature reports a variable incidence of B/SC metastases, ranging from 0.9 to 36% [5]. Within our study group, the majority of metastases of the contralateral neck were associated with concurrent ipsilateral metastases, accounting for 7.13% (30 out of 420 cases). In four instances, contralateral metastases were observed in the absence of ipsilateral involvement. Consequently, the calculated probability of developing contralateral metastases without any ipsilateral metastases was determined to be 0.95%.

In our study, higher T stage, ipsilateral LNMs, lymphatic invasion, proximity to the midline, and tumors located at the floor of the mouth or upper jaw emerged as primary factors predisposing individuals to B/SC metastases (all *p* < 0.05). Interestingly, patients with primary tumors in the upper jaw faced nearly twice the risk of B/SC metastases compared to those with tumors at the floor of the mouth. Furthermore, the risk of B/SC metastases was notably elevated in T4a tumors (38.89% compared to < 20% in T1-T3). Additionally, patients with tumors demonstrating lymphatic invasion exhibited a heightened susceptibility to B/SC metastases (19.09% vs. 39.39%). Unsurprisingly, tumors localized in the midline or bilateral were more predisposed to exhibit B/SC metastases (22.73% vs. 60.71%).

Overall, the findings of the present study are consistent with previous research, confirming that the involvement of the floor of the mouth and tongue stands as a significant prognostic factor for the development of contralateral metastases [14, 15]. Moreover, it has been observed that patients with tumors originating on the palate have an increased likelihood of developing metastases on the opposite side (20%). Thereby, several authors have advocated for bilateral ND in all cases of palate tumors with a tumor stage > 1 [5, 7, 16]. The elevated incidence of B/SC metastases observed in tumors of the upper jaw within our cohort might be attributed to the frequent multilocular presentation of tumors involving the palate and upper jaw.

The literature consistently highlights the significance of tumor size as one of the primary factors influencing the occurrence of LNMs [15, 17]. In our study, tumors classified as T3 and T4 exhibited B/SC metastases in 18.18% and 38.89% of cases, respectively. Koo et al. reported a higher incidence for T3 tumors (25%) and a notably lower probability of 18% for T4 tumors [7]. However, the decreased occurrence of B/SC metastases in T4 tumors in their study could be attributed to the size-related proximity of T3 tumors to the midline. In the present study, 14.29% of T1 tumors and 11.63% of T2 tumors exhibited B/SC metastases, consistent with findings reported in previous studies [5, 7, 14]. Olzowy et al. emphasized the overestimation of the risk of developing contralateral metastases for small tumors (T1, T2) and an underestimation for those with advanced status (T3, T4) [18].

Numerous authors recognized the pivotal role of midline involvement as a crucial factor influencing the risk of contralateral metastases [7, 19–21]. Kowalski et al. emphasized that if the tumor extends beyond the midline by more than an inch, there is an 8.8-fold higher risk of developing contralateral metastases [14]. In the current study, a total of 56 tumors extended beyond the midline, with contralateral lymph node findings observed in 34 cases (60.71%). The significant correlation (*p* = 0.011) between midline involvement and the development of contralateral metastases aligns with existing literature [5].

Capote-Moreno et al. identified ipsilateral cervical LNMs as a key predictor for the presence of LNMs on the contralateral side of the neck [6]. This finding is consistent with the outcomes of our study, wherein the presence of ipsilateral metastases significantly heightened the probability of contralateral metastases, effectively tripling the associated risk. Therefore, in clinical decision-making, the presence of ipsilateral LNMs may warrant consideration for contralateral ND.

The prognostic significance of contralateral metastases on survival rates has been explored in various studies. Iype et al. observed a 50% reduction in survival when patients presented with contralateral metastases [22]. Similarly, Capote-Moreno et al. reported a decrease in the 5-year OS from 70 to 41.2% for cases with contralateral metastases [6]. In the present study, comparison of patients with solely ipsilateral, solely contralateral, and bilateral metastases revealed poorer survival outcomes in the latter two groups. However, statistical significance was only reached in the bilateral LNMs group (log-rank, *p* = 0.010). Similarly, a significant difference was noted in OS between bilateral metastases and solely ipsilateral metastases (log-rank, *p* = 0.044). Additionally, the disparity between contralateral metastases and solely ipsilateral metastases was nearly significant (log-rank, *p* = 0.086). However, it is crucial to acknowledge that the prognosis of the patients is influenced by various factors, including surgical intervention, pathological staging, and adjuvant therapy, such as radiotherapy, chemotherapy, immunotherapy, or a combination thereof.

For therapeutic planning, the preoperative assessment of LNMs holds significant importance [23]. In our study, CT revealed bilateral metastases in 35 patients (8.31%), and contralateral metastases in 2 patients (0.48%). A certain number of patients (19 out of 420) were erroneously diagnosed with bilateral metastases, while another group (16 patients) who later showed bilateral metastases upon histological assessment were not initially identified with bilateral LNMs in CT. Malik et al. examined bilateral/contralateral metastases in oropharyngeal SCC and reported a significantly higher rate of occult B/SC metastases, reaching 9.8% [24]. Additionally, they reported a rate of 1.7% for ipsilateral clinically negative necks and 9.8% for clinically positive necks [24].

Overall, B/SC cervical metastases are rare in OSCC patients, particularly in those with a clinic-radiologically negative neck. Nevertheless, it is essential to note that in cases of therapeutic neck dissection following a “wait-and-see” approach, often referred to as “salvage ND,” there is typically a poorer prognosis [25, 26]. The adoption of a “wait-and-see” approach results in a sixfold increase in the incidence of LNMs in observation groups, leading to a significantly shortened DFS [25, 26]. Our previous investigations have indicated that occult metastases are generally no burden factor in OSCC patients when adhering to a standardized approach in ND [27]. However, the results of the present study have shown, that the small subset of patients with occult B/SC metastases exhibits notably poorer DFS compared to those without occult contralateral metastases (log-rank test, *p* = 0.040). This phenomenon may be attributed to the tumor exhibiting a more aggressive behavior when B/SC metastases occur. Nonetheless, there is no statistically significant difference in OS between patients with and without occult B/SC metastases (log-rank, *p* = 0.099).

The German guideline suggests considering END on the contralateral side in patients with higher T-stage tumors, multiple ipsilateral lymph nodes, and higher grading, especially for carcinomas located near the midline and those affecting the floor of the mouth [28]. Conversely, the American Society of Clinical Oncology (ASCO) guidelines propose considering contralateral ND or irradiation in contralateral clinically node negative patients with advanced tumors (T3/4) or when the tumor approaches the midline. They also recommend taking tumor thickness into consideration in the context of other adverse pathological factors [4]. However, the absence of high-level evidence from randomized controlled trials (RCT) hinders the establishment of a definitive standard of care in this context.

### Limitations of the study

Our study has certain limitations that warrant acknowledgment. Firstly, its retrospective and single-center design introduces inherent biases. However, it is important to acknowledge that previous studies on survival and metastases have often faced challenges due to smaller sample sizes or heterogeneous data. In this regard, our study stands out due to its substantial sample size of 420 patients and a highly homogeneous patient cohort, which excludes other types of head and neck squamous cell carcinoma, thus distinguishing it from comparable studies. Moreover, unlike previous research, we specifically investigated solely contralateral metastases, in addition to bilateral metastases. Furthermore, we uniquely examined the prevalence and implications of occult B/SC metastases in OSCC.

## Conclusion

B/SC metastases are rare in patients with OSCC, especially in those who present with clinico-radiologically negative ipsilateral necks. Higher rates of B/SC metastases are observed in cases of advanced tumors and tumors localized at the upper jaw or floor of the mouth. Ipsilateral LNMs significantly increase the likelihood of LNMs on the contralateral neck, tripling the associated risk. This insight provides valuable information for surgeons when considering whether to extend ND to the contralateral side or recommend extended adjuvant treatment. Nevertheless, the absence of high-level evidence from randomized controlled trials (RCT) hinders the establishment of a definitive standard of care in this context.

### Electronic supplementary material

Below is the link to the electronic supplementary material.

Supplementary Material 1

## Data Availability

No datasets were generated or analysed during the current study.

## Abbreviations

B/SC: bilateral/solely contralateral
CT: computed tomography
DFS: disease-free survival
DOI: depth of invasion
END: elective neck dissection
OSCC: oral squamous cell carcinoma
LNM: lymph node metastasis
ND: neck dissection
NPV: negative predictive value
OS: overall survival
PPV: positive predictive value
SND: selective neck dissection
TNM: tumor, node, metastasis

## Declarations

This study was performed in line with the principles of the 1964 Helsinki Declaration and its later amendments. The Ethics Committee of the Friedrich-Alexander University Erlangen-Nuremberg approved the study’s design and methods (Ethic votes: 23-185-Br, 23-186-Br). In accordance with national regulations and institutional regulations, written informed consent was not required from the participating patients.
