# Supplementary material for: Prevalence and implications of bilateral and solely contralateral lymph node metastases in oral squamous cell carcinoma
Source: Clin Oral Investig. 2024 Apr 23;28(5):267. doi: 10.1007/s00784-024-05650-1 (PMC11039519; doi:10.1007/s00784-024-05650-1)
Supplement: Supplementary file 1 — Supplementary Material 1 [file 784_2024_5650_MOESM1_ESM.docx]

**Supplemental material:** **Prevalence and implications of bilateral and solely contralateral lymph node metastases in oral squamous cell carcinoma**

Ann-Kristin Struckmeier MD, DMD, Mayte Buchbender DMD, Abbas Agaimy MD, Marco Kesting MD, DMD

**Table S1.** Clinicopathological characteristics of the investigated cohort

| **Characteristics** | **Number of patients (%)** |
| --- | --- |
| **No. of patients** | 420 |
| **Sex** | |
| Male | 259 (61.67) |
| Female | 161 (38.33) |
| **Age** | |
| Mean | 64.75 |
| Standard deviation | 12.03 |
| **Tumor localization** | |
| Floor of the mouth | 149 (35.48) |
| Tongue | 105 (25.00) |
| Lower jaw | 69 (16.43) |
| Upper jaw | 40 (9.52) |
| Buccal plane | 29 (6.90) |
| Palate | 22 (5.24) |
| Multilocular | 6 (1.43) |
| **Tumor stage** |  |
| T1 | 153 (36.43) |
| T2 | 108 (25.71) |
| T3 | 50 (11.90) |
| T4a | 109 (25.95) |
| **Nodal stage** |  |
| N0 | 277 (65.95) |
| N1 | 43 (10.24) |
| N2a | 11 (2.62) |
| N2b | 28 (6.67) |
| N2c | 11 (2.62) |
| N3b | 50 (11.90) |
| **Histological grading** | |
| G1 | 40 (9.52) |
| G2 | 216 (51.43) |
| G3 | 157 (37.38) |
| Gx | 4 (1.67) |
| **Lymphatic invasion** | |
| L0 | 384 (91.43) |
| L1 | 34 (8.10) |
| Lx | 2 (0.48) |
| **Vascular invasion** | |
| V0 | 409 (97.38) |
| V1 | 9 (2.14) |
| Vx | 2 (0.48) |
| **Perineural invasion** | |
| Pn0 | 336 (80.00) |
| Pn1 | 82 (19.52) |
| Pnx | 2 (0.48) |
| **Residual tumor** | |
| R0 | 409 (97.38) |
| R1 | 8 (1.90) |
| Rx | 3 (0.71) |
| **Depth of tumor invasion** |  |
| ≤ 5 mm | 175 (41.67) |
| 6-10 mm | 112 (26.67) |
| ≥ 10 mm | 100 (23.81) |
| DOIx | 33 (7.86) |
| **Type of neck dissection** |  |
| Ipsilateral SND | 159 (37.77) |
| Bilateral SND | 94 (22.33) |
| Ipsilateral MRND + Contralateral SND | 106 (25.18) |
| Bilateral MRND | 62 (14.73) |

Abbreviations: MRND = modified radical neck dissection, SND = selective neck dissection
